# Physiological and performance-related variables associated with long-distance running performance in female distance runners

**DOI:** 10.3389/fspor.2026.1877914

**Published:** 2026-07-08

**Authors:** Ryo Yamanaka, Koya Yamashiro, Daisuke Sato, Ryosuke Ando, Yasuhiro Suzuki

**Affiliations:** 1 Faculty of Human Health, Kurume University, Fukuoka, Japan; 2 Department of Health and Sports, Niigata University of Health and Welfare, Niigata, Japan; 3 Institute of Health and Sport Sciences, University of Tsukuba, Ibaraki, Japan; 4 Advanced Research Initiative for Human High Performance, University of Tsukuba, Ibaraki, Japan; 5 Department of Sports Sciences, Japan Institute of Sports Sciences, Tokyo, Japan; 6 Center for General Education, Tokyo Keizai University, Tokyo, Japan

**Keywords:** 3, 000-m running performance, OBLA, running economy, sprint performance, vLamax

## Abstract

**Background:**

Long-distance running performance is influenced by multiple physiological factors, among which aerobic capacity has traditionally been considered particularly important. Although recent evidence suggests that sprint-related characteristics also play an important role, the determinants of long-distance running performance in female athletes remain insufficiently understood.

**Purpose:**

This exploratory cross-sectional study aimed to examine the relationships between long-distance running performance and multiple physiological determinants, performance-related indices, performance-related characteristics, and physiological parameters, including aerobic capacity, running economy (RE), onset of blood lactate accumulation (OBLA), 100-m sprint time, and maximal rate of lactate accumulation (vLamax), in female long-distance runners.

**Methods:**

Thirteen trained female runners participated in this study. Physiological variables, including peak oxygen uptake (V̇O_2_peak) as an index of aerobic capacity, were assessed using treadmill-based step- and ramp-incremental tests, during which RE was defined as oxygen uptake at a running speed of 180 m·min⁻^1^. Sprint performance was evaluated using a 100-m maximal sprint test on an outdoor all-weather track.

**Results:**

Long-distance running performance was defined as the seasonal best time in the 3,000-m event (3,000-m SB: 10:07.8 ± 0:21.1). The 3,000-m SB was significantly correlated with 100-m sprint time (*r* = 0.731, *p* = 0.003), RE (*r* = 0.662, *p* = 0.010), vLamax (*r* = −0.657, *p* = 0.011), and running velocity at OBLA (*r* = −0.612, *p* = 0.020). No significant correlation was observed between 3,000-m SB and V̇O_2_peak. All significant correlations remained statistically significant after Benjamini–Hochberg false discovery rate correction.

**Conclusion:**

These findings suggest that sprint-related characteristics, RE, and lactate-related performance indices are associated with long-distance running performance in female long-distance runners.

## 1 Introduction

Long-distance running performance is influenced by the interaction of multiple physiological factors. Traditionally, maximal oxygen uptake (V̇O_2_max) (1, 2) and running economy (RE) (3, 4) have been considered key physiological determinants of endurance performance. In addition, lactate-related indices such as lactate threshold and the onset of blood lactate accumulation (OBLA) have been widely used as performance-related indicators because they reflect the integrated influence of aerobic capacity, metabolic efficiency, and the ability to sustain high exercise intensities (5, 6). The concept that endurance performance is determined by the interaction of multiple physiological characteristics, rather than by a single physiological determinant, has long been recognized in endurance performance research (7, 8). Recent work by Mougin et al. (9) has provided quantitative support for the interpretation that lactate-related running velocities should be considered integrated performance-related indices rather than independent physiological determinants. Specifically, lactate threshold- and lactate turnpoint-related velocities are largely explained by the combined influence of V̇O_2_max, RE, and the fractional utilization of V̇O_2_max (9).

Sprint performance is a performance-related characteristic reflecting the integrated influence of neuromuscular, mechanical, and metabolic factors involved in running ability. A study on male distance runners reported significant relationships between sprint performance (e.g., 100-m and 400-m sprint times) and long-distance running performance, suggesting that sprinting ability contributes to performance beyond traditional aerobic indices (10). Furthermore, resistance training interventions that increase the cross-sectional area of the psoas major muscle, which is associated with sprinting ability (11), have been shown to improve long-distance running performance (12). Moreover, a recent intervention study demonstrated that six weeks of sprinting training improved not only 100-m and 400-m sprint performance but also long-distance running performance (3,000-m running test), despite no significant changes in V̇O_2_max or oxygen cost during treadmill running (13). These findings suggest that sprint performance is associated with endurance performance beyond traditional aerobic indices. However, most of these findings are based on studies involving male athletes, and whether similar relationships exist among female distance runners remains unclear. Moreover, sex differences in physiological characteristics and physical performance have been widely reported (14), while female athletes remain underrepresented in sports science research. Therefore, it is important to examine these relationships in female distance runners.

Additionally, maximal rate of lactate accumulation (vLamax), a physiological parameter reflecting glycolytic and anaerobic metabolic capacity, has recently gained attention as a key metabolic characteristic of exercise performance (15). Although vLamax is considered an important parameter describing anaerobic metabolism, relatively few studies have examined vLamax in running performance (16). Previous studies have demonstrated a strong relationship between vLamax and 100-m sprint performance (17), and vLamax has also been shown to slightly improve the prediction of long-distance performance (18). Furthermore, vLamax and V̇O_2_max have been proposed as complementary physiological parameters reflecting the interaction between glycolytic and oxidative energy supply systems during exercise (19). A high vLamax has been considered unfavorable for endurance performance because of increased glycolytic contribution and lactate accumulation (20, 21). However, recent evidence suggests that anaerobic metabolic capacity may also contribute to performance in middle-distance events requiring both aerobic and anaerobic energy supply (15). In events such as the 3,000-m race, where aerobic and anaerobic energy systems contribute substantially, glycolytic capacity may play a role in responding to high-intensity phases of competition (22). Previous studies examining vLamax and running performance have mainly focused on sprint or middle-distance runners and broader athlete populations (15, 17, 18), with limited attention given to female distance runners. Therefore, the relationship between vLamax and performance specifically in female distance runners remains unclear.

Therefore, this exploratory cross-sectional study aimed to examine the relationships between long-distance running performance and multiple physiological determinants, performance-related indices, performance-related characteristics, and physiological parameters, including peak oxygen uptake (V̇O_2_peak), RE, OBLA, 100-m sprint time, and vLamax, in female long-distance runners.

## 2 Materials and methods

### 2.1 Participants

Thirteen female long-distance runners participated in the study. The mean ± standard deviation (SD) of the participants' age, height, and body weight were 19.5 ± 1.2 years, 158.2 ± 3.7 cm, and 48.4 ± 2.8 kg, respectively. The official average seasonal best times in 3,000-m races (3,000 m-SB) within 3 months before or after the examination were 10:07.8 ± 0:21.1. In this study, we defined 3,000 m-SB as an index of performance in long-distance runners. According to the Participant Classification Framework proposed by McKay et al. (23), all participants were classified into Tier 3 (Highly Trained/National Level). Initially, 17 female runners were scheduled to participate in the study; however, four athletes were unable to participate because of injury or illness immediately prior to testing. Therefore, data from 13 athletes were included in the final analysis. No participants who completed the testing procedures were excluded from the analyses. All participants were informed of the study's purpose and procedures verbally and in writing, and written informed consent was obtained prior to participation. The study protocol was approved by the Institutional Ethics Committee (approval number: 517).

### 2.2 Experimental design

Each participant completed two experimental sessions: (1) treadmill-based physiological testing (step- and ramp-incremental tests) and (2) a 100-m maximal sprint test. A minimum recovery period of 72 h was allowed between sessions. Participants were instructed to refrain from strenuous exercise and from caffeine and alcohol consumption for at least 24 h before each test.

### 2.3 Procedures

#### 2.3.1 Treadmill tests

Each participant performed a step-incremental test followed by a ramp-incremental test to determine V̇O_2_peak, RE, and OBLA. All the tests were conducted on a motorized treadmill at a constant inclination of 1%. After a standardized 15-min warm-up, the participants performed a step-incremental test consisting of five stages (180, 210, 240, 270, and 300 m·min^−1^). Each stage lasted 3 min, with 1 min of passive rest between stages. The step-incremental test was terminated when blood lactate concentration, measured at the end of each stage, exceeded 4 mmol·L^−1^. One minute after completing the step-incremental test, the participants performed a ramp-incremental test until exhaustion. The ramp-incremental test started at the running speed corresponding to the final completed stage of the step-incremental test, with running speed increasing by 10 m·min^−1^ every minute until volitional exhaustion.

Expired gases were continuously measured using a metabolic cart (AE-310S, Minato Medical Science, Osaka, Japan), which was calibrated before each test using a 2-L syringe and standard reference gases. Oxygen uptake was calculated as the average over 30-s intervals. V̇O_2_peak was defined as the highest oxygen uptake value obtained during the ramp-incremental test. Given that the ramp-incremental test was preceded by a step-incremental test and initiated at participant-specific running velocities, the more conservative term V̇O_2_peak was adopted rather than V̇O_2_max. RE was defined as the oxygen uptake measured at a running speed of 180 m·min^−1^.

Capillary blood samples were collected from the fingertips at the end of each stage of the step-incremental test. Blood lactate concentration was analyzed using a portable blood lactate analyzer (Lactate Pro 2, Arkray, Kyoto, Japan). The running velocity corresponding to a blood lactate concentration of 4 mmol·L^−1^ was calculated using least-squares interpolation and defined as OBLA.

#### 2.3.2 100-m maximal sprint test

The 100-m maximal sprint test was performed on an outdoor all-weather track. Sprint time was recorded to the nearest 0.01 s using photocell timing gates (SPEED TECH S-001, Xuanying, Dongguan, China). Reaction time was excluded, and net sprint time was used for the analysis.

Capillary blood samples were collected immediately before and at 1-min intervals after the sprint to determine the blood lactate concentration until two consecutive decreases in lactate values were observed. The maximal lactate accumulation rate (vLamax) was calculated using the following equation (16, 17, 24):

$$vLamax=\frac{[\mathrm{La}{]}_{\;peak}-[\mathrm{La}{]}_{\;pre}}{t_{100m}-t_{alac}}$$

where *t*_100m_ represents the 100-m sprint time, and *t*_alac_ was estimated as *t*_alac_ = *t*_100m_·0.09809 + 2.0455 (16, 17).

### 2.4 Statistical analysis

Prior to statistical analyses, normality of the data was assessed using the Shapiro–Wilk test, and all variables were confirmed to be normally distributed. All statistical analyses were performed using SPSS Statistics (version 26.0.0, IBM Corp., Armonk, NY, USA). Data are presented as mean ± SD.

An *a priori* power analysis was conducted using G*Power (version 3.1.9.7; Heinrich Heine University Düsseldorf, Germany). Assuming a large correlation effect size (*r* = 0.70), *α* = 0.05, and statistical power of 0.80, the required minimum sample size was estimated to be 13 participants. Pearson's product–moment correlation coefficients were calculated to examine the relationships between 3,000-m SB and the measured variables (RE, V̇O_2_peak, OBLA, 100-m sprint time, and vLamax). In addition, 95% confidence intervals (95% CI) for Pearson's correlation coefficients were calculated using Fisher's *z* transformation. To account for multiple comparisons, *p*-values obtained from the correlation analyses were adjusted using the Benjamini–Hochberg false discovery rate procedure. Statistical significance was set at *p* < 0.05.

A *post-hoc* sensitivity analysis was conducted using G*Power. With a sample size of 13, *α* = 0.05, and statistical power of 0.80, the minimum detectable correlation coefficient was *r* = 0.690.

## 3 Results

**Figure 1:**
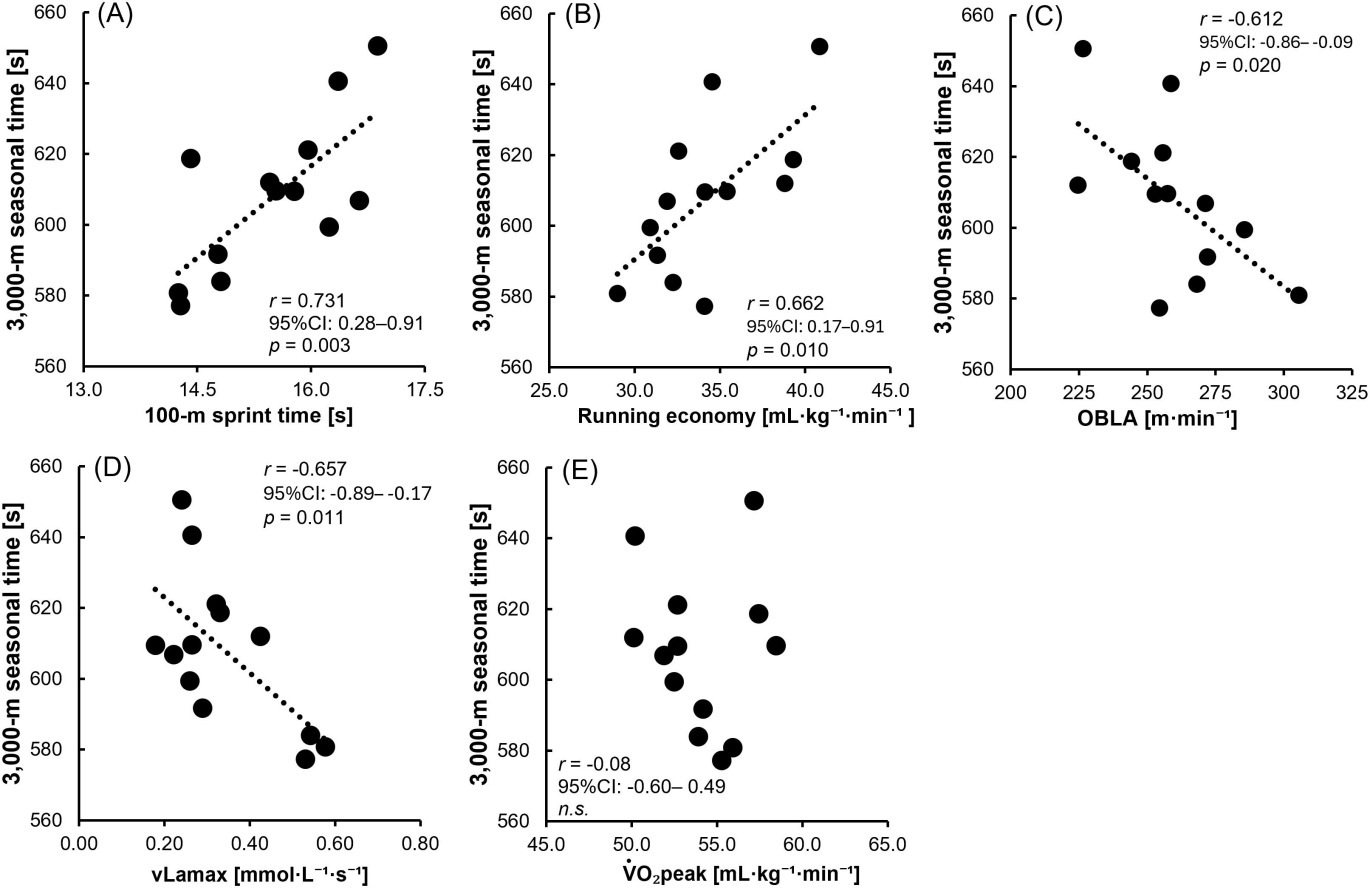
Relationships between 3,000-m seasonal best performance and the measured variables in female long-distance runners. **(A)** 100-m sprint time, **(B)** running economy (RE), **(C)** running velocity at onset of blood lactate accumulation (OBLA), **(D)** maximal rate of lactate accumulation (vLamax), and **(E)** peak oxygen uptake (V̇O_2_peak).

Table 1 presents the descriptive results for all variables. The results of the bivariate correlation analyses between 3,000-m SB and the measured variables are shown in Figure 1. The 100-m sprint time was significantly positively correlated with 3,000-m SB (*r* = 0.731, *p* = 0.003). 3,000-m SB was significantly positively correlated with RE (*r* = 0.662, *p* = 0.010). Significant negative correlations were found between 3,000-m SB and running velocity at OBLA (*r* = −0.612, *p* = 0.020). Additionally, vLamax was significantly negatively correlated with 3,000-m SB (*r* = −0.657, *p* = 0.011). In contrast, no significant correlation was observed between 3,000-m SB and V̇O_2_peak (*r* = −0.08, *p* > 0.05). All significant correlations remained statistically significant after Benjamini–Hochberg false discovery rate correction.

**Table 1:** Descriptive statistics for physiological and performance-related variables.

Variable	Mean ± SD	95%CI
3,000-m seasonal best time [s]	607.8 ± 21.1	594.6–621.1
Running economy [mL·kg^−1^·min^−1^]	34.3 ± 3.4	32.1–36.4
V̇O_2_peak [mL·kg^−1^·min^−1^]	54.0 ± 2.6	52.4–55.7
Running velocity at OBLA [m·min^−1^]	259.9 ± 21.1	246.6–273.2
100-m sprint time [s]	15.5 ± 0.9	14.9–16.0
vLamax [mmol·L^−1^·s^−1^]	0.34 ± 0.13	0.25–0.42

V̇O_2_peak, peak oxygen uptake; OBLA, onset of blood lactate accumulation (4 mmol·L^−1^); vLamax, maximal rate of lactate accumulation; 95% CI = 95% confidence interval.

The relationships between the measured variables are presented in Table 2. Significant correlations were observed between RE and OBLA (*r* = −0.918, *p* < 0.001), and between 100-m sprint time and vLamax (*r* = −0.630, *p* = 0.021). These correlations remained statistically significant after Benjamini–Hochberg false discovery rate correction. No other significant correlations were observed among the measured variables.

**Table 2:** Correlation matrix showing Pearson's correlation coefficients (r) and 95% confidence intervals (CIs).

	RE	V̇O_2_peak	OBLA	100-m sprint time
RE	–	–	–	–
V̇O_2_peak	0.229 (−0.37–0.69)	–	–	–
OBLA	−0.918** (−0.98 to −0.74)	0.007 (−0.55–0.56)	–	–
100-m sprint time	0.211 (−0.39–0.68)	−0.357 (−0.76–0.23)	−0.280 (−0.72–0.33)	–
vLamax	−0.334 (−0.75–0.27)	0.034 (−0.60–0.50)	0.317 (−0.29–0.74)	−0.630* (−0.88 to −0.13)

RE, running economy; V̇O_2_peak, peak oxygen uptake; OBLA, running velocity at onset of blood lactate accumulation (4mmol·L^−1^); vLamax, maximal rate of lactate accumulation.

* indicates *p* < 0.05.

** indicates *p* < 0.01.

## 4 Discussion

The present exploratory cross-sectional study examined the relationships between 3,000-m running performance and multiple physiological determinants, performance-related indices, performance-related characteristics, and physiological parameters, including V̇O_2_peak, RE, OBLA, 100-m sprint time, and vLamax, in female distance runners. The main findings were that 3,000-m performance was significantly associated with RE, OBLA, 100-m sprint time, and vLamax, and these associations remained statistically significant after Benjamini–Hochberg false discovery rate correction. In contrast, V̇O_2_peak was not significantly correlated with 3,000-m performance.

First, the 100-m sprint time showed a strong correlation with 3,000-m performance, and this was the strongest association among the variables examined. This finding suggests that sprint performance is closely associated with long-distance running performance, consistent with previous findings in male distance runners (10). Additionally, previous intervention studies have shown that sprint training can improve 3,000-m performance without changes in V̇O_2_max (13). Although the present study does not establish a causal relationship, these findings collectively suggest that sprint-related characteristics contribute to long-distance running performance in female long-distance runners.

Second, RE was significantly associated with 3,000-m performance. This result supports the findings of previous studies that have indicated that RE has been consistently associated with endurance performance, particularly in well-trained distance runners (4, 5). The concept that endurance performance is determined by the interaction of multiple physiological characteristics, rather than by a single physiological determinant, has long been recognized in endurance performance research (7, 8). Recent work by Mougin et al. (9) has provided quantitative support for the interpretation that lactate-related running velocities should be considered integrated performance-related indices rather than independent physiological determinants. Mougin et al. (9) reported that lactate threshold- and lactate turnpoint-related running velocities were largely explained by the combined influence of V̇O_2_max, RE, and the fractional utilization of V̇O_2_max. Therefore, the running velocity at OBLA may reflect the integrated contribution of multiple physiological characteristics associated with endurance performance rather than represent an independent physiological determinant. Although the running velocity at OBLA was significantly associated with 3,000-m performance, the magnitude of this relationship was slightly lower than that observed for RE. Consistent with this interpretation, a very strong negative correlation was observed between RE and running velocity at OBLA (*r* = −0.918). This relationship indicates that athletes with lower oxygen cost (better RE) tend to sustain higher running velocities at a given lactate level, reflecting more efficient energy utilization during submaximal and high-intensity exercise. This finding is consistent with previous studies suggesting that RE and lactate threshold are closely related, although they represent distinct physiological constructs (8, 25). Therefore, the comparatively weaker association between OBLA and 3,000-m performance may reflect substantial overlap with RE rather than a lack of physiological relevance. It should also be noted that the mean running velocity during a 3,000-m race exceeded the mean running velocity at OBLA, which is physiologically plausible because competitive 3,000-m performance operates at an intensity substantially above OBLA and is influenced by multiple physiological and performance-related characteristics (8, 9). In addition, the participant's relatively homogeneous performance level, the narrow range of V̇O_2_peak values, the use of seasonal best performances, and the small sample size may have further reduced the apparent contribution of OBLA in the present study. Therefore, the weaker association observed for OBLA should be interpreted with caution and should not be regarded as evidence that lactate-related indices are unimportant for endurance performance.

Saunders et al. (4) also reported that better RE is associated with improved endurance performance because athletes require less oxygen consumption at a given running velocity, thereby delaying metabolic fatigue. Previous studies have demonstrated that RE is strongly associated with distance running performance, particularly in highly trained runners with relatively homogeneous V̇O_2_max values (26). Additionally, it has long been suggested that improved RE contributes to the ability to maintain higher running speeds before reaching critical metabolic thresholds (27). Saunders et al. (4) summarized that RE is influenced by physiological, neuromuscular, and biomechanical factors. Furthermore, biomechanical characteristics and movement efficiency have been shown to substantially influence RE and endurance performance (28, 29). Recent studies have also suggested that RE is strongly influenced by movement efficiency and biomechanical characteristics (30). Tanji et al. (30) suggested that biomechanical characteristics associated with movement efficiency may contribute to endurance performance. These biomechanical characteristics may ultimately influence endurance performance through their effects on the metabolic cost of running. Improved RE reduces the oxygen cost of running at a given speed, thereby allowing athletes to sustain higher running velocities before reaching critical metabolic thresholds (4, 27). Although previous studies examining RE and endurance performance have predominantly involved male athletes, the present findings extend these observations to female distance runners and provide exploratory evidence that RE may also be an important performance-related factor in female athletes. The present study comprehensively evaluated multiple physiological and performance-related variables in female distance runners to explore how these factors are associated with long-distance running performance. Among these variables, RE showed a significant association with 3,000-m performance, suggesting that movement and metabolic efficiency may be important factors associated with endurance performance in female athletes. Taken together, these findings suggest that RE may represent an important performance-related factor in female distance runners, potentially reflecting the combined influence of metabolic and biomechanical efficiency. Given the exploratory nature of the present study, these findings should be interpreted cautiously.

Third, vLamax was significantly negatively associated with 3,000-m running performance in the present study. Although vLamax has traditionally been considered unfavorable for endurance performance because of increased glycolytic contribution and lactate accumulation (20), recent studies have suggested that anaerobic metabolic capacity may also contribute to performance in middle-distance events requiring both aerobic and anaerobic energy supply (15, 31). Furthermore, vLamax and V̇O_2_max have been proposed as complementary physiological parameters reflecting the interaction between glycolytic and oxidative energy supply systems during exercise (15, 19). Previous studies have demonstrated strong relationships between vLamax and sprint performance (15, 17). Consistent with these findings, a significant negative correlation was observed between 100-m sprint time and vLamax in the present study. This finding suggests that athletes with greater glycolytic capacity tend to demonstrate superior sprint performance. In events such as the 3,000-m race, where both aerobic and anaerobic energy systems contribute substantially, anaerobic metabolic characteristics may contribute to performance during high-intensity phases of competition and the final sprint (22). Previous studies analyzing elite long-distance races have demonstrated substantial increases in running velocity during the final stages of competition (32, 33), suggesting that the ability to respond to high-intensity demands may influence overall race performance. However, because 100-m sprint performance is included in the calculation of vLamax, this relationship may partly reflect a methodological association between the two variables. Therefore, the present findings regarding vLamax should be interpreted cautiously. Nevertheless, the significant association between vLamax and 3,000-m performance observed in the present study provides exploratory evidence that anaerobic metabolic characteristics may also be associated with long-distance running performance in female long-distance runners. Given the exploratory nature of the present study and the relatively small sample size, further studies are required to clarify the physiological significance of vLamax in female distance runners.

### 4.1 Limitations

This study had several limitations. First, the sample size was relatively small (*n* = 13), which may have limited the statistical power of the analyses. A *post-hoc* sensitivity analysis indicated that the present study had sufficient statistical power to detect only relatively large correlations (*r* ≥ 0.690). Therefore, smaller associations may have gone undetected. Second, the 3,000-m running performance was evaluated using seasonal best times, which were not obtained under the same conditions as the physiological measurements. Therefore, differences in environmental conditions, competition settings, and athletes' physical conditions may have influenced the results. Third, the participants were limited to highly trained female long-distance runners competing in the 3,000-m event; thus, caution should be exercised when generalizing these findings to other performance levels, events, or male athletes. Fourth, the fixed step-incremental protocol with 30 m·min^−1^ stage increments may have limited the precision of OBLA estimation in some participants. In participants who exceeded the 4 mmol·L^−1^ criterion at relatively low running speeds, only a limited number of sub-OBLA data points were available for interpolation, which may have reduced the accuracy of OBLA estimation. Fifth, the treadmill protocol used in the present study consisted of a step-incremental test followed by a ramp-incremental test with participant-specific starting velocities. Consequently, differences in exercise history and accumulated fatigue prior to the ramp test may have influenced the highest oxygen uptake values obtained. Therefore, the more conservative term V̇O_2_peak was adopted rather than V̇O_2_max, and the physiological interpretation of this variable should be made with caution. Sixth, menstrual cycle phase and hormonal fluctuations were not controlled in the present study, although these factors may influence physiological responses and athletic performance in female athletes. Seventh, because of the cross-sectional design of the present study, causal relationships between the measured variables and long-distance running performance cannot be established. Future studies with larger sample sizes and a wider range of events (e.g., 800 m, 1,500 m, 3,000 m, 5,000 m, and 10,000 m), as well as longitudinal or intervention designs, are necessary to clarify these relationships.

### 4.2 Practical applications

Our findings have important implications for training strategies for female long-distance runners. Traditionally, improvements in endurance performance have primarily focused on enhancing aerobic capacity. However, the present results suggest that sprint performance and RE are closely associated with long-distance running performance. Therefore, in addition to traditional endurance training aimed at improving aerobic fitness, incorporating short sprint training may be beneficial for developing sprint-related characteristics that are associated with long-distance running performance. Furthermore, training interventions targeting movement efficiency to improve RE may represent a useful strategy for improving endurance performance. Additionally, simple sprint measures such as 100-m sprint time may serve as practical and accessible indicators associated with performance and training adaptations. Moreover, incorporating metabolic indices such as vLamax may provide additional information regarding individual physiological characteristics, potentially facilitating more individualized training prescriptions. Nevertheless, given the exploratory nature of the present study, the relatively small sample size, and the protocol-related limitations associated with the physiological assessments, these practical implications should be confirmed in future longitudinal and intervention studies.

## 5 Conclusion

This exploratory cross-sectional study examined the relationships between 3,000-m running performance and multiple physiological determinants, performance-related indices, performance-related characteristics, and physiological parameters in female long-distance runners. The results demonstrated that 3,000-m running performance was significantly associated with RE, OBLA, 100-m sprint time, and vLamax, and these associations remained statistically significant after Benjamini–Hochberg false discovery rate correction. These findings suggest that sprint-related characteristics, RE, and lactate-related performance indices are associated with long-distance running performance in female long-distance runners. However, given the exploratory and cross-sectional nature of the present study, as well as the protocol-related limitations associated with the physiological assessments, further longitudinal and intervention studies are required to clarify the physiological mechanisms underlying these relationships.

## Data Availability

The raw data supporting the conclusions of this article will be made available by the authors, without undue reservation.
